# Tenascin-C Deficiency Is Associated With Reduced Bacterial Outgrowth During *Klebsiella pneumoniae*-Evoked Pneumosepsis in Mice

**DOI:** 10.3389/fimmu.2021.600979

**Published:** 2021-03-11

**Authors:** Mariska T. Meijer, Alex F. de Vos, Brendon P. Scicluna, Joris J. Roelofs, Chérine Abou Fayçal, Gertraud Orend, Fabrice Uhel, Tom van der Poll

**Affiliations:** ^1^Center for Experimental and Molecular Medicine, Amsterdam University Medical Centers, Location Academic Medical Center, University of Amsterdam, Amsterdam, Netherlands; ^2^Amsterdam Institute for Infection and Immunity, Amsterdam University Medical Centers, Amsterdam, Netherlands; ^3^Clinical Epidemiology Biostatistics and Bioinformatics, Amsterdam University Medical Centers, Location Academic Medical Center, University of Amsterdam, Amsterdam, Netherlands; ^4^Department of Pathology, Amsterdam University Medical Centers, Location Academic Medical Center, University of Amsterdam, Amsterdam, Netherlands; ^5^The Tumor Microenvironment Laboratory, INSERM UMR_S 1109, Université Strasbourg, Faculté de Médecine, Hopital Civil, Institut d'Hématologie et d'Immunologie, Fédération de Médecine Translationnelle de Strasbourg (FMTS), Strasbourg, France; ^6^Division of Infectious Diseases, Amsterdam University Medical Centers, Location Academic Medical Center, University of Amsterdam, Amsterdam, Netherlands

**Keywords:** tenascin C, sepsis, *Klebsiella pneumoniae* (*K. pneumoniae*), pneumonia, alarmins, innate immunity, immune system, mice

## Abstract

Tenascin C (TNC) is an extracellular matrix glycoprotein that recently emerged as an immunomodulator. TNC-deficient (TNC^−/−^) mice were reported to have a reduced inflammatory response upon systemic administration of lipopolysaccharide, the toxic component of gram-negative bacteria. Here, we investigated the role of TNC during gram-negative pneumonia derived sepsis. TNC^+/+^ and TNC^−/−^ mice were infected with *Klebsiella pneumoniae* via the airways and sacrificed 24 and 42 h thereafter for further analysis. Pulmonary TNC protein levels were elevated 42 h after infection in TNC^+/+^ mice and remained undetectable in TNC^−/−^ mice. TNC^−/−^ mice showed modestly lower bacterial loads in lungs and blood, and a somewhat reduced local—but not systemic—inflammatory response. Moreover, TNC^−/−^ and TNC^+/+^ mice did not differ with regard to neutrophil recruitment, lung pathology or plasma markers of distal organ injury. These results suggest that while TNC shapes the immune response during lipopolysaccharide-induced inflammation, this role may be superseded during pneumosepsis caused by a common gram-negative pathogen.

## Introduction

Tenascin C (TNC) is a large multimeric extracellular matrix glycoprotein with binding sites for many different signaling factors. It plays a major role during embryonic development, but in adult tissues expression levels are relatively low (1, 2). However, TNC is expressed in a variety of cell types in response to both chemical and mechanical cellular stress (1, 2). Recently, TNC has drawn attention for its immunomodulatory properties (3–6).

Midwood and colleagues were the first to show that in a model of rheumatoid arthritis, inflammation could not be maintained beyond the first 24 h when mice were TNC deficient (3). Despite displaying a normal acute immune response, the Tenascin C deficient (TNC^−/−^) mice did not develop the chronic inflammation displayed by TNC sufficient (TNC^+/+^) mice. Moreover, it was reported that TNC can act as a direct agonist of toll-like receptor 4 (TLR4), which also serves as the main signaling receptor for lipopolysaccharide (LPS), a proinflammatory component of the gram-negative bacterial cell wall (4, 7). Thus, TNC was proposed as a putative danger associated molecular peptide (DAMP). In agreement with a role of TNC as a pro-inflammatory signaling molecule, treatment with TNC siRNA suppressed LPS-induced cytokine production by mouse macrophages (8). In accordance, TNC^−/−^ mice became less severely ill after LPS injection, which was associated with decreased tumor necrosis factor (TNF)-α and interleukin (IL)-6 production (7). Together these data suggest that TNC may enhance acute and chronic inflammation.

We and others have previously shown that TNC plasma levels are increased in critically ill patients, particularly in those suffering from infection and sepsis (9, 10). Sepsis patients represent 10% of all admissions to the intensive care unit (ICU), and sepsis is the leading cause of disease in non-coronary ICUs in the developed world with a mortality rate of 20–30%. Its pathophysiology is the result of a dysregulated immune response with concurrent hyperinflammation and immune suppression (11). The most common cause of sepsis is bacterial pneumonia, and the Gram-negative bacterium *Klebsiella (K.) pneumoniae* is frequently isolated from septic patients (12). Host defense to *K. pneumoniae* pneumonia largely depends on TLR4 (13, 14). In the present study we therefore sought to determine if TNC regulates the host response during pneumonia derived sepsis caused by *K. pneumoniae*. To this end, we performed *in vivo* experiments causing pneumonia and sepsis in TNC^−/−^ and TNC^+/+^ mice by infection with *K. pneumoniae* via the airways.

## Materials and Methods

### Ethical Statement

The experiments were reviewed and approved by the Institutional Animal Care and Use Committee of the Academic Medical Center (AMC), University of Amsterdam (identification numbers DIX21-EV-1 and DIX288-BP-1). The animal care and use protocol adhered to European Directive of 22 September 2010 (Directive 2010/63/EU) in addition to the Directive of 6 May 2009 (Directive 2009/41/EC).

### Animals

TNC^+/+^ and TNC^−/−^ mice, bred on a C57BL/6 background for 10 generations (15), were bred in parallel from heterozygous parents. Mice were housed in individually ventilated cages enriched with disposable homes and nesting paper, and provided with food and water *at libitum*. All mice were bred and housed at the Animal Research Institute AMC under specific pathogen free conditions. Mice were acclimatized in the procedure room for at least one week before commencement of the experiment. Mice entered experiments at 8–10 weeks of age and in good health. Both genders were used for experiments and groups were sex matched. Mice were assessed on their welfare (including posture and activity) throughout their stay at the facility.

### Study Design

Experimental groups consisted of 8 (24-h time point) or 12 (42-h time point) mice per genotype. This corresponds to a power of 80%, type I error of 5%, standard deviation of 35%, and effect sizes of 50% (24 h) or 35% (42 h). Home cages were placed in a random order, which was then used throughout the experiment. Pneumonia was induced by intranasal administration of 10^4^ colony-forming units (CFU) of *K. pneumoniae* serotype 2 (ATCC 43816) in 50 μL sterile isotonic saline as described (13, 16). In brief, bacteria were grown for 2–3 h to a mid-logarithmic phase at 37°C using Tryptic Soy broth, harvested by centrifugation and resuspended in sterile isotonic saline so that 50 μL corresponded to 10^4^ CFU. Inoculation was performed under sedation with 2–3% isoflurane in 100% O_2_ to ensure calm inspiration of the inoculum. Mice were euthanized at 24 or 42 h after inoculation by intraperitoneal injection of ketamine (125 mg/kg) and dexmedetomidine (300 μg/kg) followed by cardiac puncture. Blood was collected into heparin tubes (Microtainer, BD Biosciences, NJ). Lung, spleen, and liver were harvested and homogenized in sterile saline (weight:volume, 1:5) using a tissue homogenizer (Biospec Products, Bastlesville, OK). CFU in organ homogenates and blood were determined from serial dilutions plated on blood agar plates incubated at 37°C for 14 h (13). Unless specified otherwise, data was collected from all animals.

### Cytokine Quantification

Plasma TNF-α, IL-6, and chemokine (C-C motif) ligand 2 (CCL2) were measured using Cytometric Bead Array (Mouse Inflammation Kit; BD Biosciences, Franklin Lakes, NJ), according to manufacturer's instructions on a FACS Canto II with High Throughput Sampler (BD, Biosciences, Franklin Lakes, NJ). Aspartate aminotransferase (AST), alanine aminotransferase (ALT), and lactate dehydrogenase (LDH) were measured by using a c702 Roche Diagnostics (Roche Diagnostics BV, Almere, the Netherlands) (17).

To determine levels of cytokine and neutrophil products in lung tissue, homogenized lung samples were diluted with an equal volume of lysis buffer (pH 7.4) containing 1% Triton X-100, 100 mM NaCl, 15 mM Tris, 1 mM MgCl_2_, 1 mM CaCl_2_ and complete™ Protease Inhibitor Cocktail (Roche, Basel, Switzerland), and incubated at 4°C for 30 min. Homogenates were centrifuged at 1500×g at 4°C for 15 min, and supernatants were stored at−20°C until analysis. Pulmonary TNC, as well as lung cytokines, chemokines and neutrophil products were then measured by ELISA according to the instructions of the manufacturers: TNC (large isoform containing FNIII-C, IBL International, Hamburg, Germany), TNF-α (Thermo Fisher Scientific, Waltham, MA), IL-1β, IL-6, chemokine (C-X-C motif) ligand (CXCL)1, CXCL2, myeloperoxidase (MPO) and neutrophil elastase (R&D Systems, Minneapolis, MN).

In addition to protein measurements, the mRNA expression levels of 5 cytokines were determined through quantitative polymerase chain reaction (qPCR). RNA was isolated from long homogenate using the Nucleospin RNA isolation kit according to manufacturer's protocol (Macherey-Nagel, Düren, Germany). cDNA was synthesized using M-MLV Reverse Transcriptase with oligo(dT) primers according to manufacturer's protocol (Promega, Madigon, WI). mRNA levels were determined through quantitative polymerase chain reaction using the SensiFast^TM^ No-ROX Kit (Bioline, London, UK) measured on a LightCycler 480 (Roche, Basel, Switzerland) using the following primers: TNF-α forward: CGAGTGACAAGCCTGTAGCC, TNF-α reverse: CCTTGAAGAGAACCTGGGAGT, IL-1β forward: GGGGAACTCTGCAGACTCAA, IL-1β reverse: GGGCCTCAAAGGAAAGAATC, IL-6 forward: CTTCCTACCCCAATTTCCAATGCT, IL-6 reverse: TCTTGGTCCTTAGCCACTCCTT, CXCL1 forward: CCACTGCACCCAAACCGAAG, CXCL1 reverse: TCCGTTACTTGGGGACACCT, mCXCL2 forward: CACTCTCAAGGGCGGTCAA and CXCL2 reverse: TCTTTGGTTCTTCCGTTGAGG. As a housekeeping gene, HPRT1 was measured using the following primers: HRPT1 forward: AGTCAAGGGCATATCCAACA and HPRT1 reverse: CAAACTTTGCTTTCCGGGT.

### Histopathology

Lungs were collected and fixed in 10% formalin in PBS for at least 16 h, transferred to 70% ethanol and embedded in paraffin. Four-micrometer paraffin-embedded lung sections were stained with hematoxylin and eosin. Slides were coded and lung inflammation and damage was scored by a pathologist blinded for group identity. To score lung inflammation and damage, the entire lung surface was analyzed with respect to the following parameters: interstitial inflammation, edema, endothelialitis, bronchitis, and pleuritis. Each parameter was graded on a scale of 0–4, (0: absent; 1: mild; 2: moderate; 3: severe; 4: very severe). The percentage pneumonia was scored and graded on a scale of 0–5 (0: absent; 1: 5–20% confluent pneumonia; 2: 21–40%; 3: 41–60%; 4: 61–80%; 5: 81–100%). Lastly, the number of thrombi were counted. All parameters and corresponding scores, as present in our dataset, our illustrated in Supplementary Figure 1. The pathology score was expressed as the sum of the scores for each parameter (16).

### Immunohistochemistry

To visualize TNC protein in the lung, paraffin-embedded lung sections were unmasked by boiling in a 10 mM pH 6 citrate solution. Slides were blocked with 5% normal goat serum in PBS and incubated with 1:100 primary anti-TNC antibody (AB19011, EMD Millipore, Temecula, CA). Slides were washed with 0.1% triton X-100 in PBS (Sigma-Aldrich, St Louis, MO) followed by 1:200 secondary anti-rabbit biotinylated antibody (Jackson ImmunoResearch, West Grove, PA). Slides were washed with 0.1% triton X-100 in PBS and treated with peroxide solution [0.6% H_2_O_2_ (Sigma-Aldrich, St Louis, MO) in methanol]. Slides were washed with 0.1% triton X-100 in PBS then were incubated with 3,3′-Diaminobenzidine developing solution (Vector Lab, Burlingame, CA) for 1 h at room temperature. The signal detection was done with the Elite ABC system (Vectastain, Burlingame, CA) and hematoxylin staining was performed. The sections were embedded into ProLong Gold antifade reagent (Invitrogen, Waltham, MA). Sections were examined using an Axio Imager A1. Pictures were taken with an AxioCam Icc3 camera and Axiovision software (all: Zeiss, Jena, Germany).

### Statistical Analysis

Statistical analysis was performed in R 3.6.3 (18). Figures were created with ggplot2 3.3.0 (19). Data was log_10_-transformed for analysis. All data presented are back-transformed through their antilog. Tables and bar graphs show means with standard errors (SE). Figures show Tukey box-and-whisker plots, including median and interquartile range without outliers. Differences between groups over time were tested using two-way type III ANOVA, which takes into account imbalanced designs. *Post-hoc* tests were performed using Tukey's HSD. Two-group comparisons were performed using an unpaired *t*-test. Data below the lower limit of detection was imputed at the lower limit of detection. A *p*-value < 0.05 was considered statistically significant.

## Results

### Pulmonary Tenascin C Modestly Impairs Antibacterial Defense During *Klebsiella* Pneumonia

**Figure 1:**
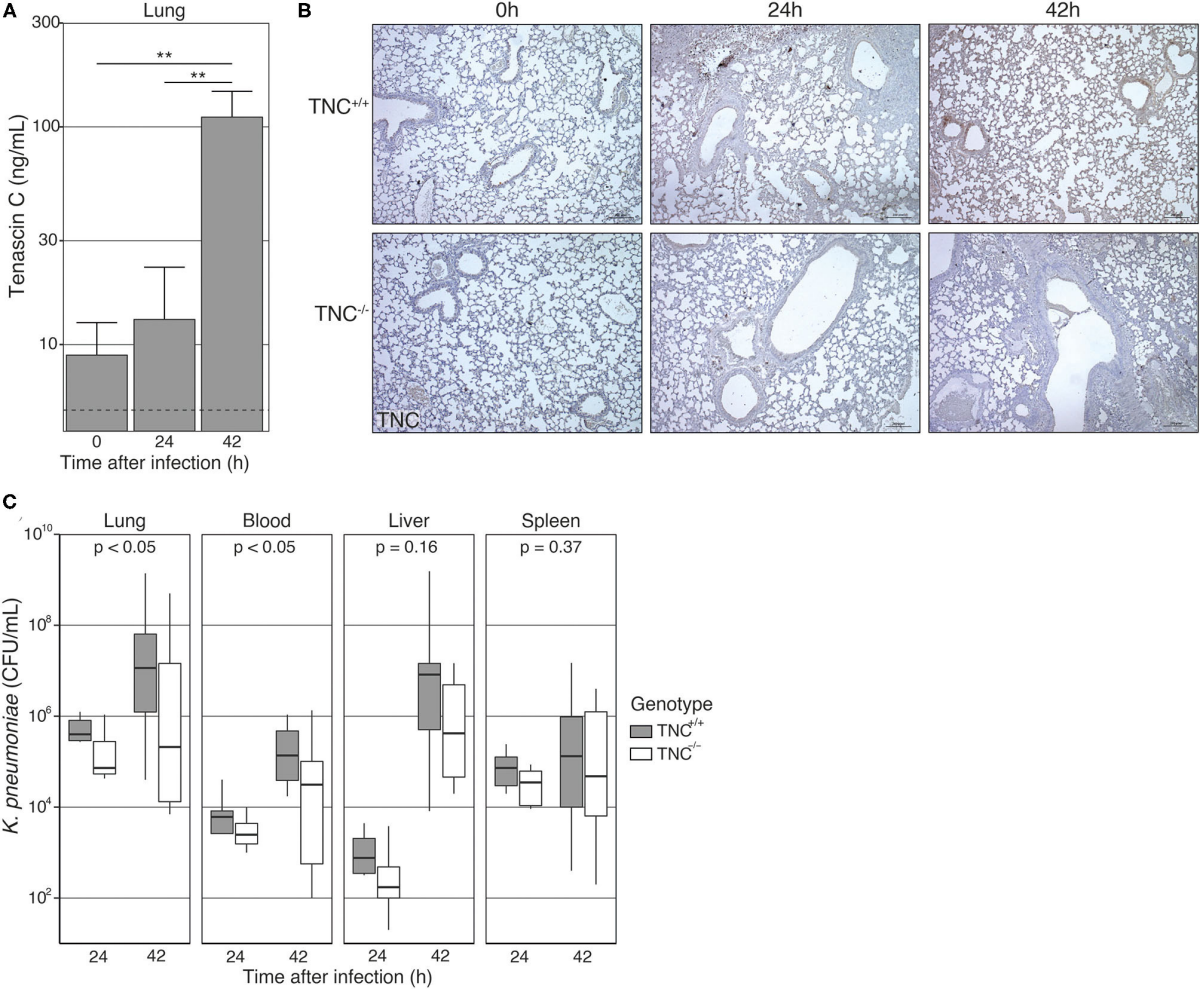
Pulmonary Tenascin-C levels and bacterial counts during *Klebsiella* induced pneumosepsis. Tenascin C sufficient (TNC^+/+^) and deficient (TNC^−/−^) mice were intranasally infected with *K. pneumoniae*. **(A)** Before inoculation, and 24 and 42 h thereafter lung samples were collected and homogenized. TNC was measured in the lysate of 4 TNC^+/+^ mice from each time point. Bars and whiskers show mean and SE. The dotted line represents the lower limit of quantitation. TNC was not detectable in any of the TNC^−/−^ samples measured. **(B)** Lung tissue was collected, fixed and embedded in paraffin 24 and 42 h after infection, as well as in the naïve state. Tissue slides were stained for TNC protein. Depicted slides are representative of 5 independent biological replicates. **(C)** Number of colony-forming units (CFU) 24 and 42 h after infection. Data are shown as Tukey boxplots without outliers. **p < 0.01 in an unpaired *t*-test. *P*-values **(C)** represent the effect of genotype across time-points, as indicated by a two-way type III ANOVA.

TNC was detected at relatively high levels in lung homogenates of naïve TNC^+/+^ mice [8.9 (3.0-26.8) ng/mL; Figure 1A], but not of TNC^−/−^ mice, documenting constitutive presence of this protein in the lungs. Infection with *K. pneumoniae* via the airways resulted in a gradual rise in lung TNC levels that at 42 h was more than 10-fold over those measured in naïve mice [42 h: 110.8 (46.5-264.1) ng/mL, *p* < 0.01]. In contrast, plasma levels of TNC did not change during infection with *K. pneumonia* [42 hours: 1443 (1069-1948) ng/mL] compared to uninfected mice [1457 (975-2178) ng/mL, *p* = 0.95]. TNC was not detected in plasma from TNC^−/−^ mice. Staining lung tissue for TNC confirmed the presence of TNC protein in the pulmonary extracellular matrix of TNC^+/+^ mice after infection, while TNC^−/−^ tissue remained negative (Figure 1B).

To determine the role of TNC in the host response during *Klebsiella* pneumonia derived sepsis we assessed bacterial burdens at the primary site of infection (lungs) and distant body sites (blood, liver and spleen) in TNC^+/+^ and TNC^−/−^ mice 24 and 42 h after infection (Figure 1C). Overall, bacterial loads were slightly lower in TNC^−/−^ than in TNC^+/+^ mice, which reached statistical significance for lung and blood (Figure 1C).

### The Absence of Tenascin C Modestly Reduces Lung Inflammation During *Klebsiella* Pneumonia

**Figure 2:**
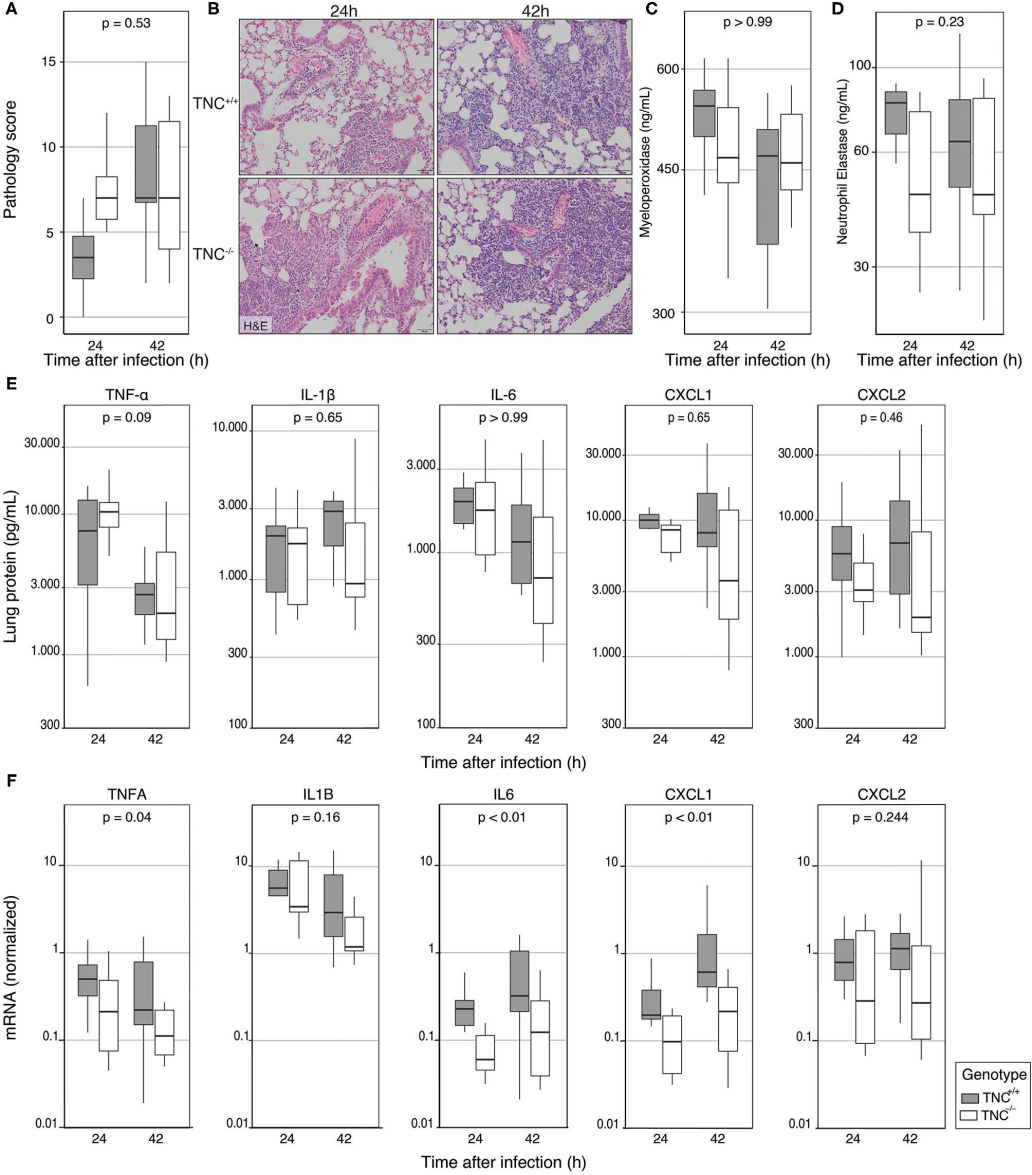
Endogenous Tenascin-C does not impact lung inflammation during *Klebsiella* induced pneumosepsis. TNC^+/+^ and TNC^−/−^ mice were intranasally infected with *K. pneumoniae;* 24 and 42 h after infection, lung tissue samples were collected for histological examination or homogenization. **(A)** Lung samples of all mice were scored for interstitial inflammation, edema, endothelialitis, bronchitis, pleuritis, and the presence of thrombi by a blinded pathologist blinded for the group identity, after which the total pathology score was calculated. **(B)** Representative pictures of lung pathology at 24 h (middle panel) and 42 hours (right panel) after infection in TNC^+/+^ mice (top panel) and TNC^−/−^ mice (bottom panel). **(C)** Levels of MPO, **(D)** elastase, and **(E)** cytokines and chemokines were measured in lung homogenates. **(F)** Cytokine mRNA was measured in RNA isolated from lung homogenate, and is presented normalized to HPRT1 expression. Data are shown as Tukey boxplots without outliers. *p*-values represent the effect of genotype across time-points, as indicated by a two-way type III ANOVA.

TNC has been implicated as an important mediator of inflammatory responses (1, 3, 4, 7). In light of the high constitutive pulmonary TNC levels and the strong induction of TNC in the lungs during *Klebsiella* pneumonia, we considered it of interest to determine the role of TNC in lung inflammation induced by this infection. To this end, we scored lung tissue slides prepared from TNC^+/+^ and TNC^−/−^ mice 24 and 42 h after induction of infection. Overall, the extent of lung inflammation did not differ between mouse strains, although TNC^−/−^ mice tended to have higher pathology scores at 24 h (Figures 2A,B).

The levels of neutrophil products MPO (Figure 2C) and elastase (Figure 2D) in lung homogenates did not differ between TNC^+/+^ and TNC^−/−^ mice, indicating similar neutrophil influx in the lung. Analysis of pulmonary cytokines (TNF-α, IL-1β, IL-6) and chemokines (CXCL1, CXCL2) revealed no significant differences between the groups (Figure 2E). However, at the mRNA level we did observe a decrease in cytokine expression, which reached significance for TNF-α, IL-6 and CXCL-1 (Figure 2F).

### The Absence of Tenascin C Does Not Influence Systemic Inflammation During *Klebsiella* Pneumonia

**Figure 3:**
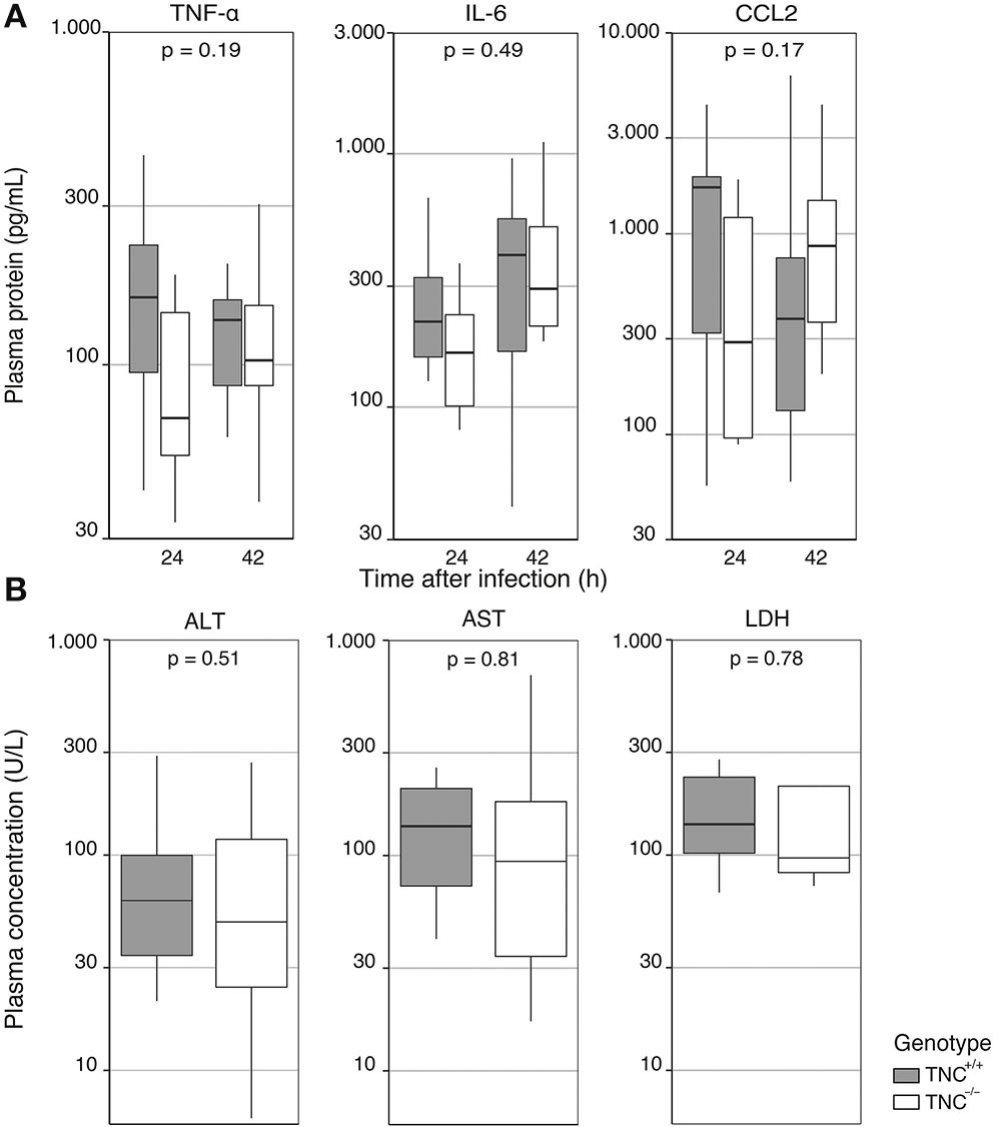
Endogenous Tenascin-C does not impact systemic inflammation during *Klebsiella* induced pneumosepsis. TNC^+/+^ and deficient TNC^−/−^ mice were infected with *K. pneumoniae;* **(A)** 24 and 42 h after infection plasma was collected for measurements of TNF-α, IL-6, and CCL2. **(B)** Markers of hepatic injury (ALT, AST) and cell injury in general (LDH) were measured at 42 h. Data are shown as Tukey boxplots without outliers. *p*-values represent the effect of genotype across time-points, as indicated by a two-way type III ANOVA **(A)**, or unpaired *t*-test **(B)**.

Patients with sepsis have elevated circulating levels of TNC (9, 10) and TNC may contribute to the pro-inflammatory and injurious systemic host response in this condition (1). The model of *Klebsiella* induced pneumosepsis is associated with systemic inflammation and distant organ injury (17). To determine the role of TNC in the development of systemic inflammation, we measured the plasma levels of TNF-α, IL-6, and CCL2 as markers of systemic inflammation 24 and 42 h after the beginning of infection (Figure 3A). Systemic cytokine levels did not differ between the groups. In addition, we measured the plasma concentrations of ALT, AST and LDH as markers of hepatocellular and general cell injury (Figure 3B); none of these injury markers differed between TNC^+/+^ and TNC^−/−^ mice.

## Discussion

Here we investigated the hypothesis that TNC may play a role in the enhancement of inflammatory responses during sepsis. This assumption was based on multiple observations. First, TNC plasma levels are elevated in patients with sepsis. Second, TNC can induce proinflammatory cytokines by activation of TLR4 in multiple cell types, including macrophages and dendritic cells, and conversely, macrophages with reduced or no TNC expression display reduced proinflammatory cytokine production upon stimulation with LPS *in vitro* (1, 2, 7, 8). Third, TNC^−/−^ mice showed attenuated proinflammatory cytokine release upon injection of LPS and the inflammation-enhancing role of TNC is further supported by mouse studies reporting a protective anti-inflammatory effect of TNC deficiency in a number of inflammatory disease models, including hepatic ischemia/reperfusion injury (20), concanavalin A–induced hepatitis (21), joint inflammation (3, 22), autoimmune encephalomyelitis (23), and Alzheimer's disease (24). Moreover, TNC has been shown to play essential proinflammatory roles in models of lung inflammation, such as ovalbumin-induced asthma (25) and bleomycin-induced pulmonary fibrosis (26, 27). As such, increased TNC levels, induced as a result of tissue damage, cellular stress, and inflammatory mediators, would be expected to be part of a positive feedback loop that amplifies the inflammatory response. However, while we detected a rise in TNC levels in lungs after airway infection with viable *K. pneumoniae*, the role of TNC in local and systemic inflammatory responses elicited by this common sepsis pathogen was very limited. Indeed, although TNC^−/−^ mice showed a modest reduction in bacterial outgrowth in lungs and blood, this preceded the rise of pulmonary TNC protein levels. We found a reduced expression of TNF-α, IL-6, and CXCL1 mRNA in the lung at both timepoints. A similar trend could be seen at the protein level, although this did not reach significance.

Inhibition of TNC expression in mouse macrophages was reported to result in attenuated LPS-induced TNF-α and IL-6 release *in vitro* (8). Likewise, TNC^−/−^ bone-marrow derived macrophages produced less TNF-α, IL-6 and CXLC1, and more IL-10 in response to LPS stimulation *in vitro* and TNC^−/−^ mice produced less TNF-α and IL-6 shortly after systemic LPS administration *in vivo* (7). These observations were confirmed using human primary material, as reviewed in (2). We observed a similar effect at the mRNA level in lungs. At the protein level, a trend toward reduced IL-6 and CXCL1 did not reach significance, possibly because of the high variability within groups. The modest differences in bacterial loads may have influenced the extent of inflammation; vice versa differences in inflammatory responses may impact antibacterial defense mechanisms. Studies with killed bacteria or bacterial products such as LPS can provide insight in the role of TNC during non-infectious lung inflammation. As the cytokine protein levels were measured in lung homogenate, they reflect the sum of intracellular, transmembrane, and secreted protein, which could contribute to the increased variance. This holds especially for TNF-α and IL-1β, which are detected in their unprocessed cell-associated forms (before cleavage by ADAM17 and caspase-1, respectively), as well as in their secreted forms. Measurements of cytokines in bronchoalveolar lavage fluid will provide insight into secretion of mature proteins into the bronchoalveolar space, but not in tissue levels. Notably, several studies in which mediators were measured in both bronchoalveolar lavage fluid and lung homogenates reported similar results, although levels in lavage fluid were consistently lower due to the dilution factor introduced by the lavage procedure (28–32). Sampling of bronchoalveolar lavage fluid can generate information on the role of TNC in cell recruitment into the airways, which in the current study was limited to examination of lung tissue slides. Protein levels of TNF-α and IL-1β in lung homogenates may be more representative of the inflammatory potential than of active cytokine signaling. Nonetheless, it has been shown that *in vitro*, TNC post-transcriptionally regulates TNF-α production of BMDMs via the micro-RNA miR-155, and this function could not be rescued through addition of extracellular TNC protein (7). This opens up the possibility that TNC plays an intracellular role in the innate immune system—and thus its absence may affect the innate immune response even before the tissue levels of TNC protein rise in TNC-sufficient animals. Further studies into the early innate immune response during pulmonary infection, as well as the *TNC* mRNA expression in innate immune cells at this time may further illuminate the underlying mechanisms.

Interestingly, Uddin et al. (8) show a 4-fold upregulation in serum TNC protein levels, already after 4 h of LPS injection, whereas we find no change in serum TNC and an increase in pulmonary TNC protein only after 42 h. Future studies into the early timepoints of *K. pneumoniae*-induced pneumonia could reveal if there is an early, transient peak in TNC serum levels. However, in this context, it is important to note that in our model, progression of inflammation during infection occurs over a longer time and causes the host to be exposed to a mix of pathogenic proteins, compared to an endotoxemia model where a known dose of LPS is directly injected into the peritoneum (7, 8). Thus, the two models describe a different type of immune response, during which TNC may play different roles. Indeed, LPS injection provides a standardized stimulus not subjected to variation in time due to differences in bacterial growth. While mice from different genotypes were infected with the exact same inoculum for studies at each time point, differences in bacterial loads might partially obscure the role of TNC in inflammatory responses. Finally, it should be noted that most *in vivo* work on the immunomodulatory properties of TNC was done in cells and mice on a 129/Sv genetic background (7), while we used mice on a C57/BL6 genetic background; these mouse strains differ in their immune response (33). 129/Sv mice were recently reported to lack caspase-11, which is expected to impact their responsiveness to LPS (34, 35).

A recent *in vivo* study described that the absence of TNC subtly alters the morphology of the lungs and affects transforming growth factor-β and TLR4 signaling, again in the 129/Sv background (36). In our study we did not find a difference in morphological pathology caused by infection. It has recently been reviewed that the role of TNC is highly context-dependent, and while it is often associated with an enhanced immune response and increased tissue damage, TNC can also play an immune-suppressive role depending on both the inflammatory context and the splicoform expressed (37). Thus, subtle changes in pulmonary morphology and signaling could alter the microenvironment and affect bacterial proliferation and dissemination, as well as the form and function of the TNC protein. It was recently described that the biosynthesis of TNC protein, counting over 500 splicoforms, highly varies between cell types as well as the (patho)biological context (38). The different splicoforms can vary greatly in their structure, function and location—indicating that they may regulate the microenvironment in different manners depending on the cell and context in which they were produced (38). In the current manuscript, TNC protein was quantified only in the Large FN(III)C isoform. Future studies aiming to quantify specific TNC isoforms with known immunomodulatory properties may provide more detailed information on the immune modulation of TNC during infection.

While pulmonary TNC protein levels increased at the latest stages of infection, histological staining did not reveal the source of this protein. Previous studies have indicated that immune cells are able to secrete TNC, which could then enhance inflammation in an autocrine manner (4). However, TNC expression levels remained low, and seem insufficient to explain the total increase of TNC protein observed in this study. In models of viral inflammation, poly(I:C) stimulation of TLR3 induced TNC release by bronchial epithelial cells, both *in vitro* and *in vivo* (39). However, we observed an increase in TNC protein levels only during the latest stages of infection, when both tissue injury and inflammation have become systemic. While this could be due to the time required for *de novo* synthesis and secretion of TNC, an extra-pulmonary source of TNC protein cannot be excluded, since TNC can be expressed by a wide variety of cell types in response to cellular stress (1, 2). Nonetheless, this would not explain why TNC levels would increase in the pulmonary compartment, but not in circulation. Future studies may try to elucidate the exact source of TNC protein during pulmonary infection. In addition, studies from the field of virology indicate TNC can interact directly with pathogens. For example, TNC in breastmilk binds HIV-1, and therewith neutralizes it (40). In contrast, the bacterial staphylococcal superantigen-like protein binds TNC in a manner that disrupts keratinocyte function during wound healing (41). As, in the present study, the absence of TNC affects bacterial outgrowth without large modulations to the immune system, it may be of interest to study if there is a direct interaction between the bacteria and TNC protein. Alternatively, modulations to the immune system may be very local, or affect cell populations that have not been studied in this context, such as innate lymphoid cells. Future studies into the inflammatory microenvironment might address these questions. Additionally, sampling of bronchoalveolar lavage fluid could generate information on the role of TNC in cell recruitment into the airways, which in the current study was limited to examination of lung tissue slides.

The current study is the first to investigate the functional role of TNC in experimental sepsis. The model used here resembles the clinical scenario of a local bacterial infection that subsequently disseminates to distant organs and has been utilized to obtain insight into both the early protective innate immune response and the later detrimental consequences of exaggerated inflammation (13, 16, 17). While this investigation is limited to a single model and a single causative pathogen, and the absence of TNC could be compensated through different pathways, the results reported here argue against an important role for total TNC protein as a driver in the early pathogenesis of sepsis. However, the heterogeneous function of different TNC splicoforms warrants more detailed studies that address the paradoxical nature of this protein.

## Data Availability Statement

The raw data supporting the conclusions of this article will be made available by the authors, without undue reservation.

## Ethics Statement

The animal study was reviewed and approved by Institutional Animal Care and Use Committee Academic Medical Center (AMC), University of Amsterdam.

## Author Contributions

MM: conceptualization, analysis, investigation, methodology, funding acquisition, writing–original draft, writing–review, and editing. AV: conceptualization, methodology, supervision, writing–review, and editing. BS and FU: methodology, supervision, writing–review, and editing. JR and CA: investigation, methodology, writing–review, and editing. GO: methodology, writing–review, and editing. TP: conceptualization, methodology, supervision, funding acquisition, writing–review, and editing. All authors contributed to the article and approved the submitted version.

## Conflict of Interest

The authors declare that the research was conducted in the absence of any commercial or financial relationships that could be construed as a potential conflict of interest.
