# Supplementary material for: Tenascin-C Deficiency Is Associated With Reduced Bacterial Outgrowth During Klebsiella pneumoniae-Evoked Pneumosepsis in Mice
Source: Front Immunol. 2021 Mar 11;12:600979. doi: 10.3389/fimmu.2021.600979 (PMC7990887; doi:10.3389/fimmu.2021.600979)
Supplement: Supplementary file 1 [file Image_1.pdf]

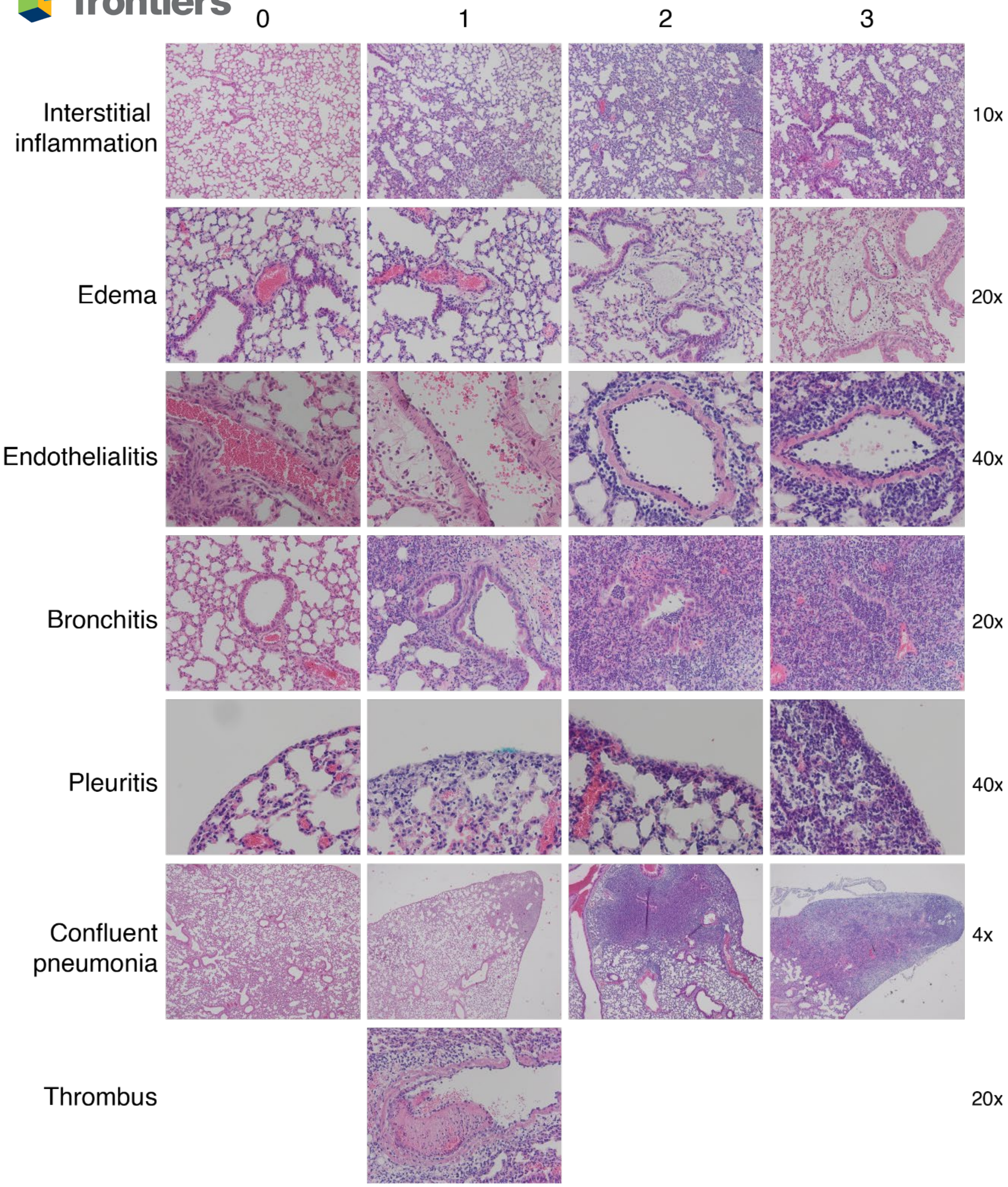

Supplementary Figure 1. As described in the methods section, lungs from all mice were collected, fixed and stained with hematoxylin and eosin. To assess lung inflammation and damage, slides were then scored by a pathologist for interstitial inflammation, edema, endothelialitis, bronchitis and pleuritis. All parameters were scored on a scale from 0–4, (0: absent; 1: mild; 2: moderate; 3: severe; 4: very severe). In addition, confluent pneumonia was scored on a scale of 0-5 (0: absent; 1: 5–20% confluent pneumonia; 2: 21–40%; 3: 41–60%; 4: 61–80%; 5: 81–100%). Panels, from left to right, show lungs that were scored as 0, 1, 2 or 3 in the current study. Rows represent the different parameters indicated on the left-hand side. Numbers on the right-hand side indicate the enlargement at which the images were taken. The final row shows the presence a thrombus. For these, the total number per lung were counted and included in the pathology score.
